# Supplementary material for: Differential Effects of Anti-TNFα and Anti-α4β7 Drugs on Circulating Dendritic Cells Migratory Capacity in Inflammatory Bowel Disease
Source: Biomedicines. 2022 Aug 4;10(8):1885. doi: 10.3390/biomedicines10081885 (PMC9405461; doi:10.3390/biomedicines10081885)
Supplement: Supplementary file 1 [file biomedicines-10-01885-s001.zip › biomedicines-1795991-supplementary.pptx]

## Slide 1
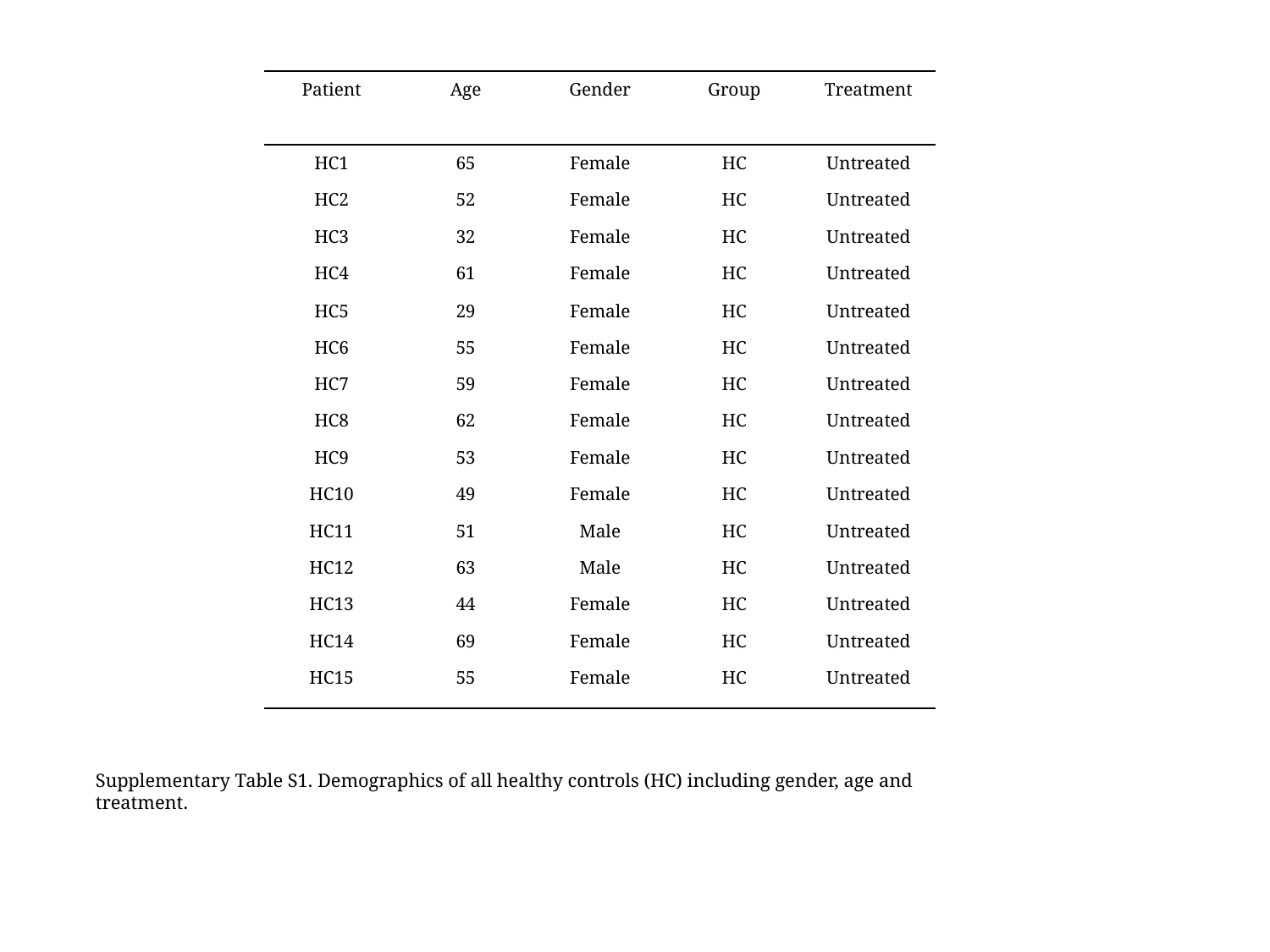

| Patient | Age | Gender | Group | Treatment |
| --- | --- | --- | --- | --- |
| | | | | |
| HC1 | 65 | Female | HC | Untreated |
| HC2 | 52 | Female | HC | Untreated |
| HC3 | 32 | Female | HC | Untreated |
| HC4 | 61 | Female | HC | Untreated |
| HC5 | 29 | Female | HC | Untreated |
| HC6 | 55 | Female | HC | Untreated |
| HC7 | 59 | Female | HC | Untreated |
| HC8 | 62 | Female | HC | Untreated |
| HC9 | 53 | Female | HC | Untreated |
| HC10 | 49 | Female | HC | Untreated |
| HC11 | 51 | Male | HC | Untreated |
| HC12 | 63 | Male | HC | Untreated |
| HC13 | 44 | Female | HC | Untreated |
| HC14 | 69 | Female | HC | Untreated |
| HC15 | 55 | Female | HC | Untreated |
Supplementary Table S1. Demographics of all healthy controls (HC) including gender, age and treatment.

## Slide 2
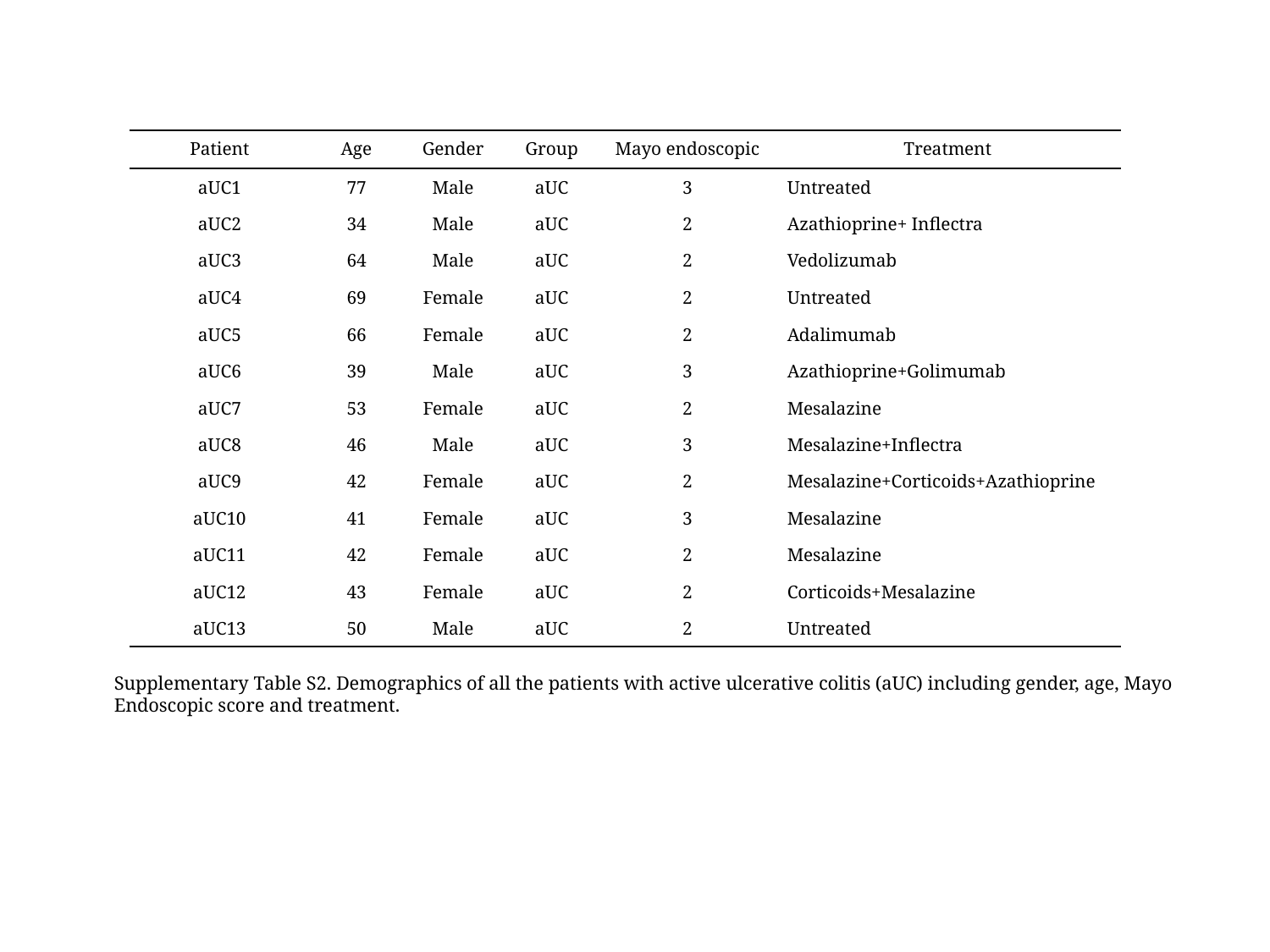

| Patient | Age | Gender | Group | Mayo endoscopic | Treatment |
| --- | --- | --- | --- | --- | --- |
| aUC1 | 77 | Male | aUC | 3 | Untreated |
| aUC2 | 34 | Male | aUC | 2 | Azathioprine+ Inflectra |
| aUC3 | 64 | Male | aUC | 2 | Vedolizumab |
| aUC4 | 69 | Female | aUC | 2 | Untreated |
| aUC5 | 66 | Female | aUC | 2 | Adalimumab |
| aUC6 | 39 | Male | aUC | 3 | Azathioprine+Golimumab |
| aUC7 | 53 | Female | aUC | 2 | Mesalazine |
| aUC8 | 46 | Male | aUC | 3 | Mesalazine+Inflectra |
| aUC9 | 42 | Female | aUC | 2 | Mesalazine+Corticoids+Azathioprine |
| aUC10 | 41 | Female | aUC | 3 | Mesalazine |
| aUC11 | 42 | Female | aUC | 2 | Mesalazine |
| aUC12 | 43 | Female | aUC | 2 | Corticoids+Mesalazine |
| aUC13 | 50 | Male | aUC | 2 | Untreated |
Supplementary Table S2. Demographics of all the patients with active ulcerative colitis (aUC) including gender, age, Mayo Endoscopic score and treatment.

## Slide 3
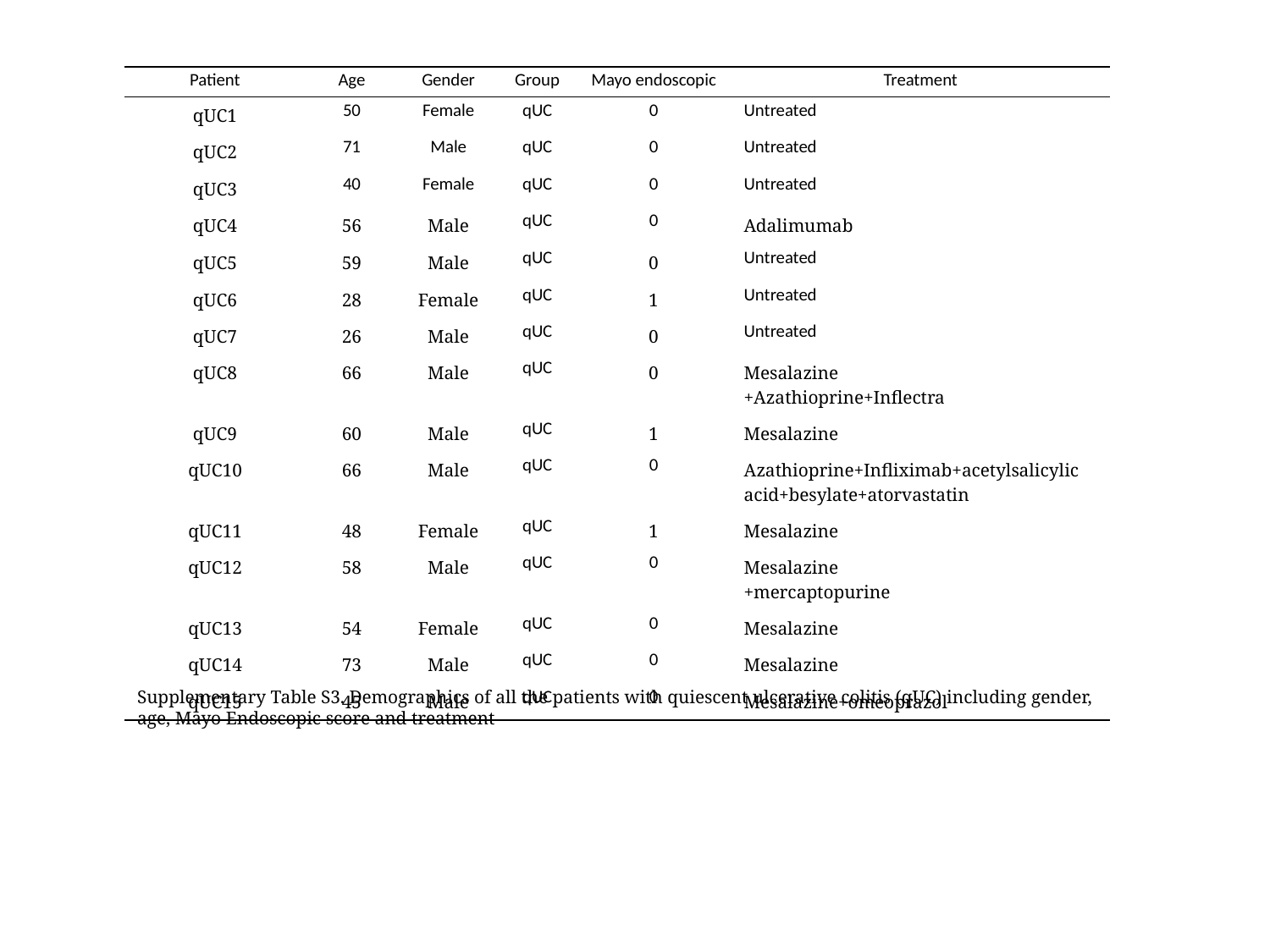

| Patient | Age | Gender | Group | Mayo endoscopic | Treatment |
| --- | --- | --- | --- | --- | --- |
| qUC1 | 50 | Female | qUC | 0 | Untreated |
| qUC2 | 71 | Male | qUC | 0 | Untreated |
| qUC3 | 40 | Female | qUC | 0 | Untreated |
| qUC4 | 56 | Male | qUC | 0 | Adalimumab |
| qUC5 | 59 | Male | qUC | 0 | Untreated |
| qUC6 | 28 | Female | qUC | 1 | Untreated |
| qUC7 | 26 | Male | qUC | 0 | Untreated |
| qUC8 | 66 | Male | qUC | 0 | Mesalazine +Azathioprine+Inflectra |
| qUC9 | 60 | Male | qUC | 1 | Mesalazine |
| qUC10 | 66 | Male | qUC | 0 | Azathioprine+Infliximab+acetylsalicylic acid+besylate+atorvastatin |
| qUC11 | 48 | Female | qUC | 1 | Mesalazine |
| qUC12 | 58 | Male | qUC | 0 | Mesalazine +mercaptopurine |
| qUC13 | 54 | Female | qUC | 0 | Mesalazine |
| qUC14 | 73 | Male | qUC | 0 | Mesalazine |
| qUC15 | 45 | Male | qUC | 0 | Mesalazine+omeoprazol |
Supplementary Table S3. Demographics of all the patients with quiescent ulcerative colitis (qUC) including gender, age, Mayo Endoscopic score and treatment

## Slide 4
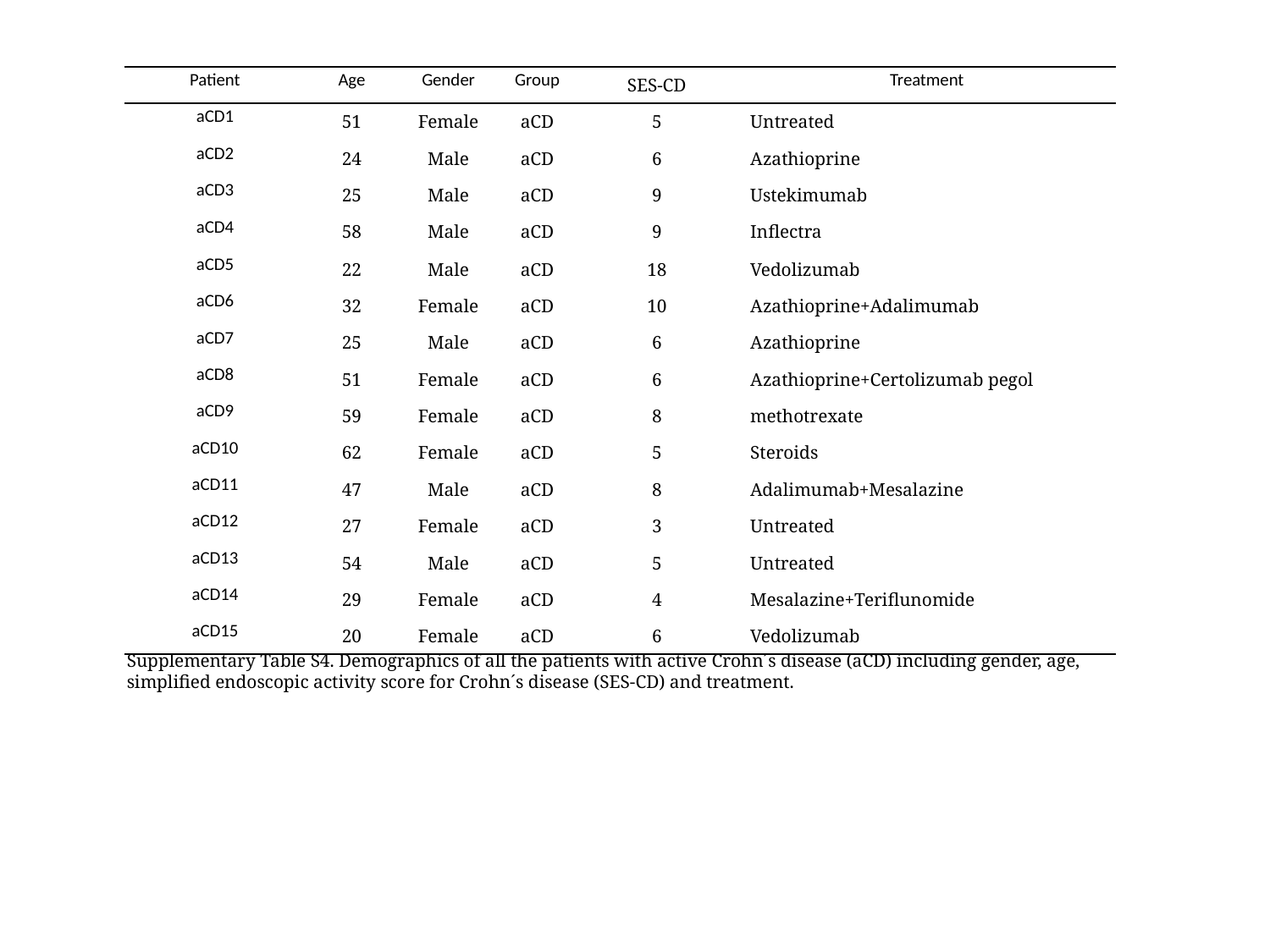

| Patient | Age | Gender | Group | SES-CD | Treatment |
| --- | --- | --- | --- | --- | --- |
| aCD1 | 51 | Female | aCD | 5 | Untreated |
| aCD2 | 24 | Male | aCD | 6 | Azathioprine |
| aCD3 | 25 | Male | aCD | 9 | Ustekimumab |
| aCD4 | 58 | Male | aCD | 9 | Inflectra |
| aCD5 | 22 | Male | aCD | 18 | Vedolizumab |
| aCD6 | 32 | Female | aCD | 10 | Azathioprine+Adalimumab |
| aCD7 | 25 | Male | aCD | 6 | Azathioprine |
| aCD8 | 51 | Female | aCD | 6 | Azathioprine+Certolizumab pegol |
| aCD9 | 59 | Female | aCD | 8 | methotrexate |
| aCD10 | 62 | Female | aCD | 5 | Steroids |
| aCD11 | 47 | Male | aCD | 8 | Adalimumab+Mesalazine |
| aCD12 | 27 | Female | aCD | 3 | Untreated |
| aCD13 | 54 | Male | aCD | 5 | Untreated |
| aCD14 | 29 | Female | aCD | 4 | Mesalazine+Teriflunomide |
| aCD15 | 20 | Female | aCD | 6 | Vedolizumab |
Supplementary Table S4. Demographics of all the patients with active Crohn´s disease (aCD) including gender, age, simplified endoscopic activity score for Crohn´s disease (SES-CD) and treatment.

## Slide 5
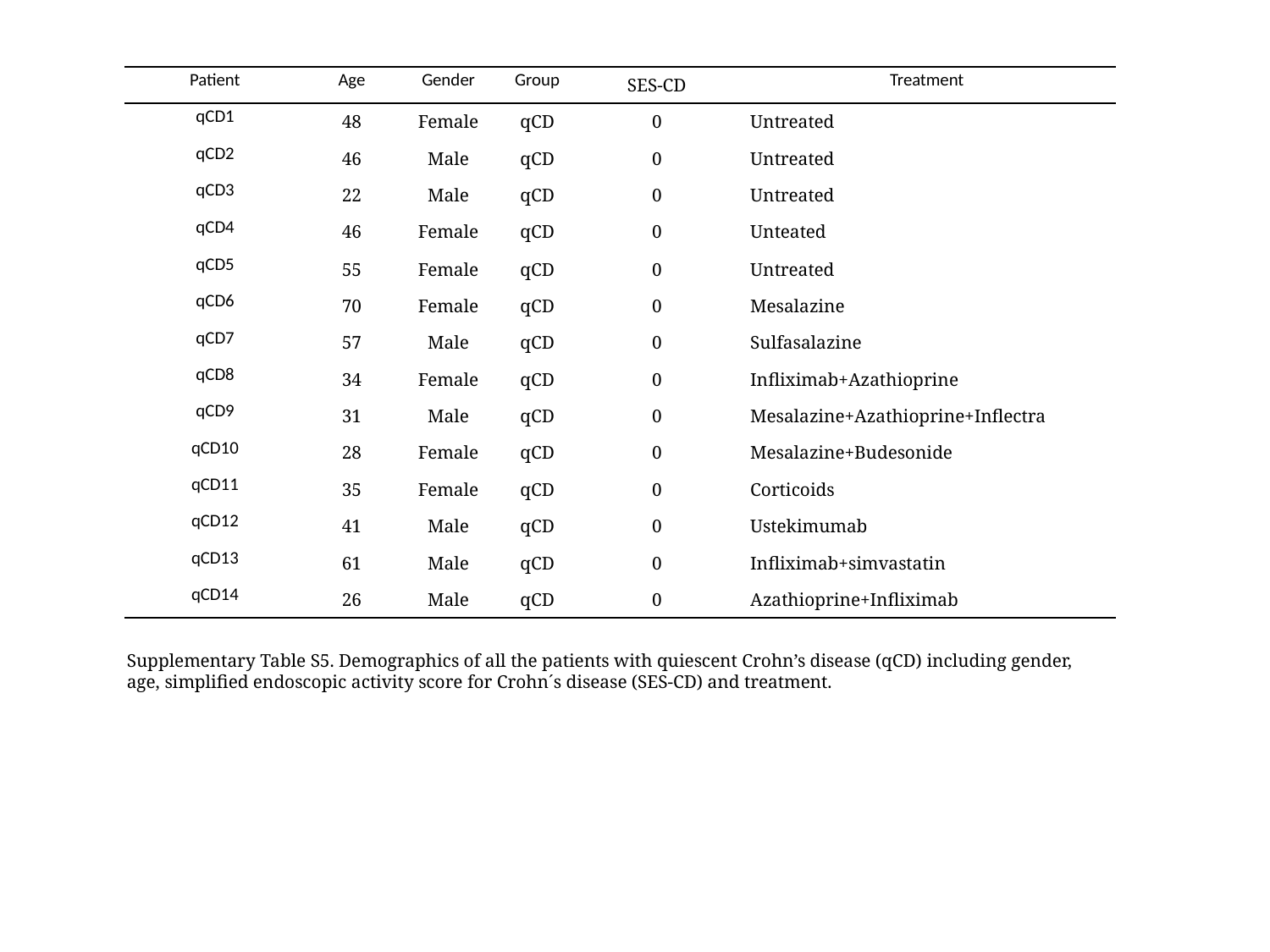

| Patient | Age | Gender | Group | SES-CD | Treatment |
| --- | --- | --- | --- | --- | --- |
| qCD1 | 48 | Female | qCD | 0 | Untreated |
| qCD2 | 46 | Male | qCD | 0 | Untreated |
| qCD3 | 22 | Male | qCD | 0 | Untreated |
| qCD4 | 46 | Female | qCD | 0 | Unteated |
| qCD5 | 55 | Female | qCD | 0 | Untreated |
| qCD6 | 70 | Female | qCD | 0 | Mesalazine |
| qCD7 | 57 | Male | qCD | 0 | Sulfasalazine |
| qCD8 | 34 | Female | qCD | 0 | Infliximab+Azathioprine |
| qCD9 | 31 | Male | qCD | 0 | Mesalazine+Azathioprine+Inflectra |
| qCD10 | 28 | Female | qCD | 0 | Mesalazine+Budesonide |
| qCD11 | 35 | Female | qCD | 0 | Corticoids |
| qCD12 | 41 | Male | qCD | 0 | Ustekimumab |
| qCD13 | 61 | Male | qCD | 0 | Infliximab+simvastatin |
| qCD14 | 26 | Male | qCD | 0 | Azathioprine+Infliximab |
Supplementary Table S5. Demographics of all the patients with quiescent Crohn’s disease (qCD) including gender, age, simplified endoscopic activity score for Crohn´s disease (SES-CD) and treatment.

## Slide 6
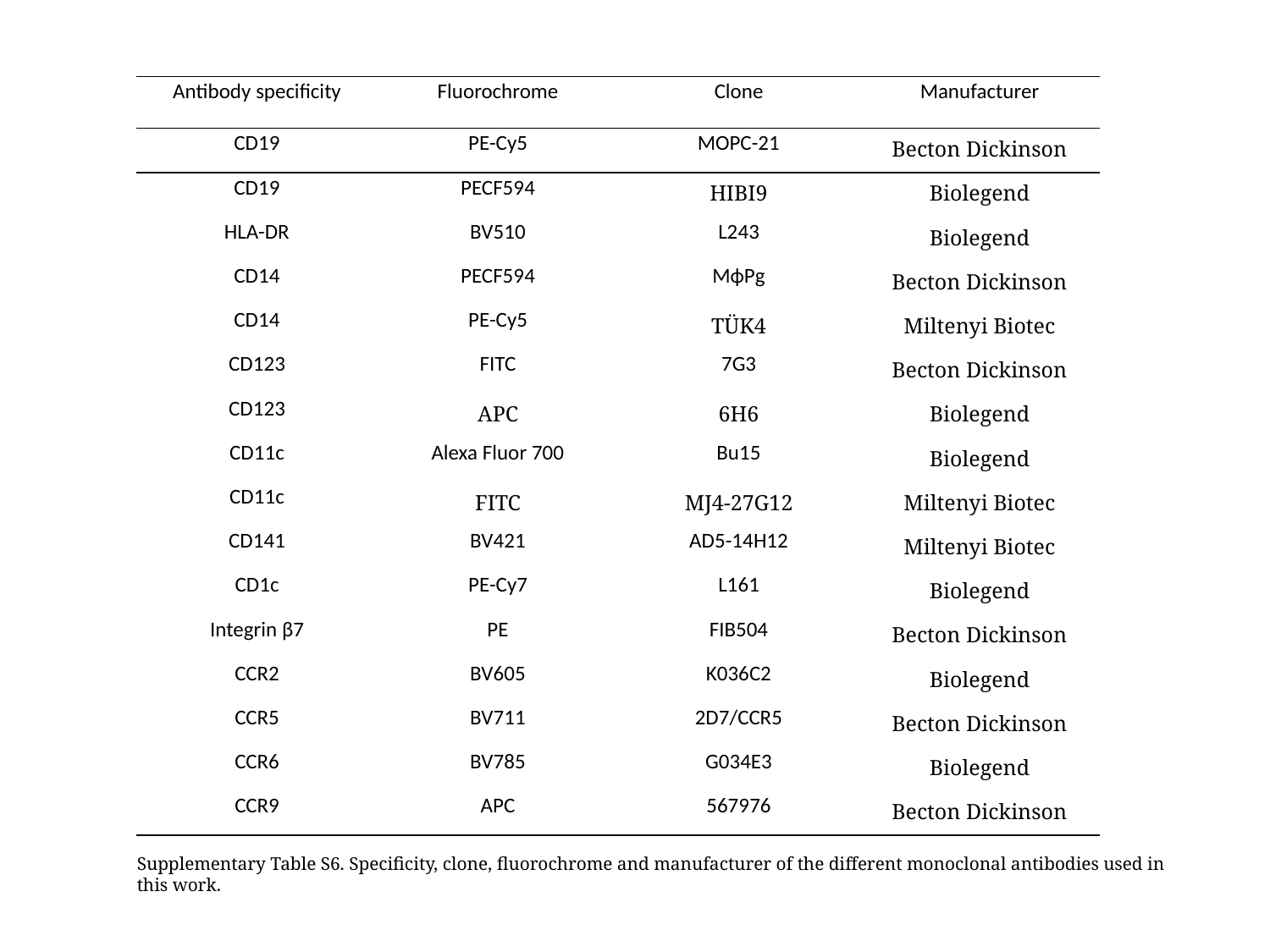

| Antibody specificity | Fluorochrome | Clone | Manufacturer |
| --- | --- | --- | --- |
| CD19 | PE-Cy5 | MOPC-21 | Becton Dickinson |
| CD19 | PECF594 | HIBI9 | Biolegend |
| HLA-DR | BV510 | L243 | Biolegend |
| CD14 | PECF594 | MϕPg | Becton Dickinson |
| CD14 | PE-Cy5 | TÜK4 | Miltenyi Biotec |
| CD123 | FITC | 7G3 | Becton Dickinson |
| CD123 | APC | 6H6 | Biolegend |
| CD11c | Alexa Fluor 700 | Bu15 | Biolegend |
| CD11c | FITC | MJ4-27G12 | Miltenyi Biotec |
| CD141 | BV421 | AD5-14H12 | Miltenyi Biotec |
| CD1c | PE-Cy7 | L161 | Biolegend |
| Integrin β7 | PE | FIB504 | Becton Dickinson |
| CCR2 | BV605 | K036C2 | Biolegend |
| CCR5 | BV711 | 2D7/CCR5 | Becton Dickinson |
| CCR6 | BV785 | G034E3 | Biolegend |
| CCR9 | APC | 567976 | Becton Dickinson |
Supplementary Table S6. Specificity, clone, fluorochrome and manufacturer of the different monoclonal antibodies used in this work.

## Slide 7
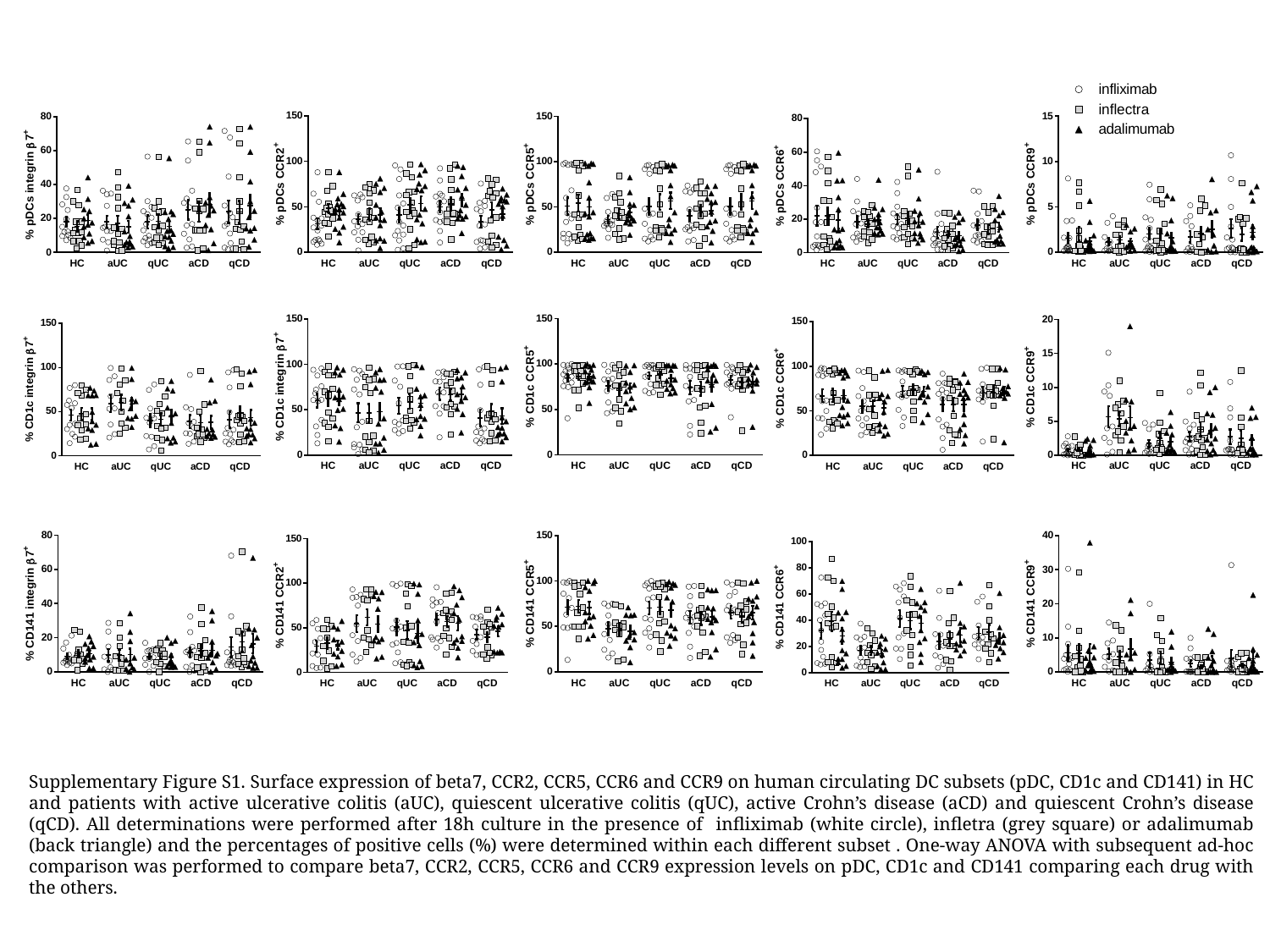

Supplementary Figure S1. Surface expression of beta7, CCR2, CCR5, CCR6 and CCR9 on human circulating DC subsets (pDC, CD1c and CD141) in HC and patients with active ulcerative colitis (aUC), quiescent ulcerative colitis (qUC), active Crohn’s disease (aCD) and quiescent Crohn’s disease (qCD). All determinations were performed after 18h culture in the presence of infliximab (white circle), infletra (grey square) or adalimumab (back triangle) and the percentages of positive cells (%) were determined within each different subset . One-way ANOVA with subsequent ad-hoc comparison was performed to compare beta7, CCR2, CCR5, CCR6 and CCR9 expression levels on pDC, CD1c and CD141 comparing each drug with the others.

## Slide 8
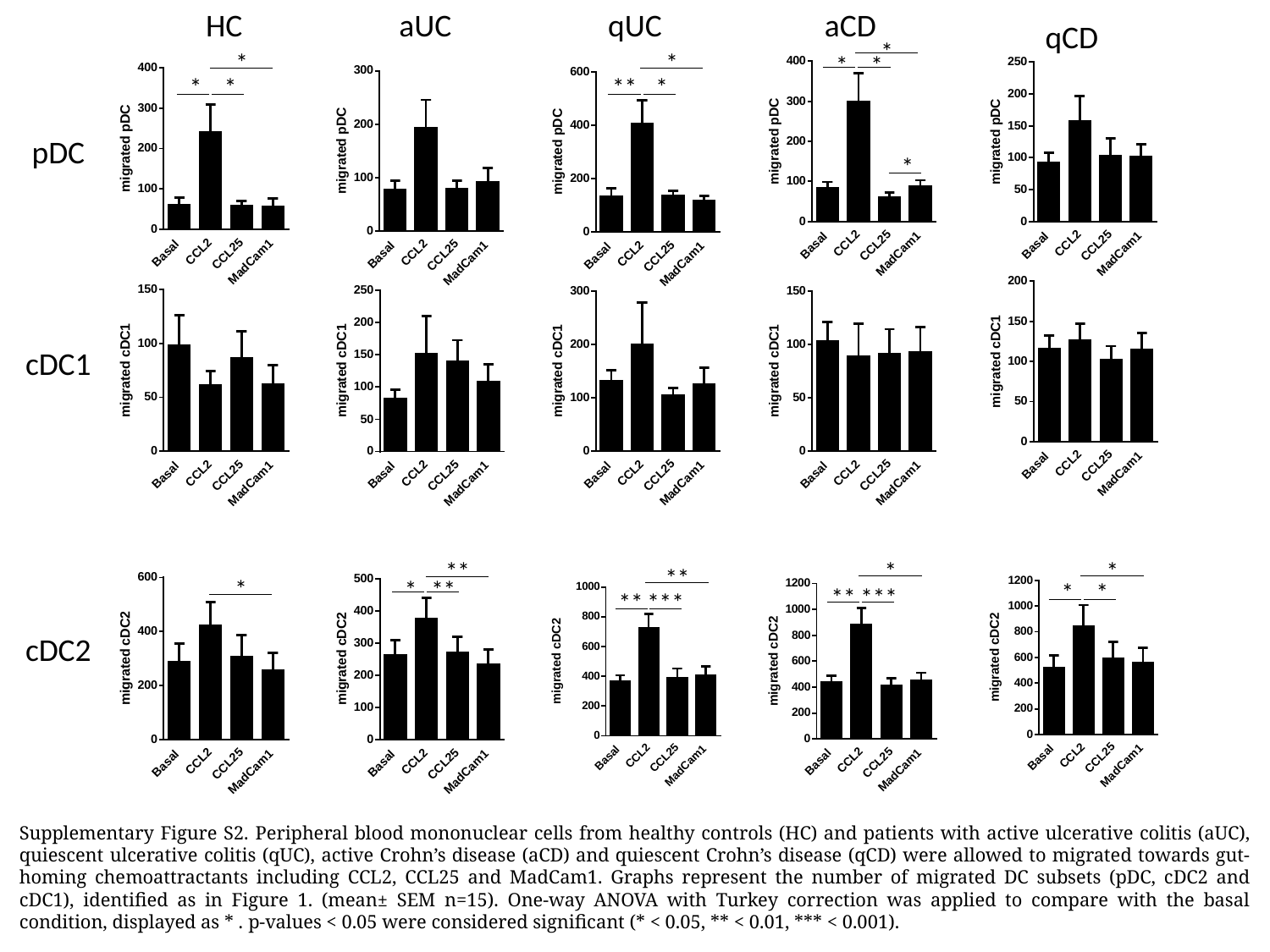

HC
aUC
qUC
aCD
qCD
*
*
*
**
*
*
*
*
*
pDC
*
cDC1
*
*
**
**
*
*
**
*
*
**
***
**
***
cDC2
Supplementary Figure S2. Peripheral blood mononuclear cells from healthy controls (HC) and patients with active ulcerative colitis (aUC), quiescent ulcerative colitis (qUC), active Crohn’s disease (aCD) and quiescent Crohn’s disease (qCD) were allowed to migrated towards gut-homing chemoattractants including CCL2, CCL25 and MadCam1. Graphs represent the number of migrated DC subsets (pDC, cDC2 and cDC1), identified as in Figure 1. (mean± SEM n=15). One-way ANOVA with Turkey correction was applied to compare with the basal condition, displayed as * . p-values < 0.05 were considered significant (* < 0.05, ** < 0.01, *** < 0.001).
